# From Here to Eternity—The Theory and Practice of a Really Long Experiment

**DOI:** 10.1371/journal.pbio.1002185

**Published:** 2015-06-23

**Authors:** Jeremy W. Fox, Richard E. Lenski

**Affiliations:** 1 Department of Biological Sciences, University of Calgary, Calgary, Canada; 2 Department of Microbiology and Molecular Genetics, Michigan State University, East Lansing, Michigan, United States of America

## Abstract

In February 1988, Richard Lenski set up 12 replicate populations of a single genotype of *Escherichia coli* in a simple nutrient medium. He has been following their evolution ever since. Here, Lenski answers provocative questions from Jeremy Fox about his iconic "Long-Term Evolution Experiment" (LTEE). The LTEE is a remarkable case study of the interplay of determinism and chance in evolution—and in the conduct of science.

## Abstract 2

Richard Lenski has been running his long-term evolution experiment for over 27 years. In this interview, ecologist Jeremy Fox asks him how and why he does it.

## Editor’s Introduction

More than 27 years ago, Richard Lenski set up his Long-Term Evolution Experiment (LTEE), in which 12 replicate populations of the workhorse bacterium *Escherichia coli* were placed into simple, identical environments with glucose as the limiting resource. The experiment may sound easy in principle, but the practical implications are immense; every day since February 24, 1988, a member of Lenski’s lab has taken 1% of each culture and diluted the cells into fresh media. And every 75th day (500 generations), someone has frozen the remainder of each population as a fossil record of the accumulated changes.

The evolving bacterial populations, now more than 60,000 generations into this marathon experiment, have provided fruit for many influential studies, and the advent of new technologies, inconceivable in 1988, has ensured that the LTEE continues to yield surprises.

This February, the University of Calgary celebrated Darwin Day with a public talk by Lenski, and local ecologist Jeremy Fox marked the occasion with questions for Lenski about the LTEE and related aspects of his career.

His questions reflect the fact that the LTEE is interesting not only because of the scientific results that have emerged from it but also because of its status as a case study of how to do science. Most touch on issues relevant to anyone who has had to decide what scientific question to ask and how to go about answering it. Is my project too high-risk? Or too low-risk? Is my study system too simple to be meaningful? What are my hypotheses? (If any!) What actually counts as an experiment? How can I fund it? The resulting conversation played out on Fox’s “Dynamic Ecology” blog and Lenski’s blog, “Telliamed Revisited” (blog URLs are also provided at the end of this article). What follows is a condensed version of that conversation. – Roland Roberts

## Conversation

**Jeremy Fox (JF):** Rich, when you started the LTEE [Long-Term Evolution Experiment], did you consider it a low-risk or high-risk experiment? I could see arguing both ways. It’s low risk, because one can imagine lots of possible outcomes, all of which would be interesting if they occurred. But in other ways, it’s high risk—I imagine that many of the interesting outcomes (including those that occurred) would’ve seemed unlikely, if indeed you’d imagined them at all when you started. Or did you not worry much about the range of possible outcomes because the experiment was basically a lottery ticket? “This’ll be cheap and not much work, let’s just do it and see what happens.”

**Richard Lenski (RL):** Life was good, and I wasn’t thinking about risk. Or as they say about investing, it’s better to be lucky than smart!

I’d already had success with some shorter duration, more traditionally designed experiments, and so it wasn’t a total shot in the dark—I knew the LTEE would yield data. I also knew, though, it was an unusually abstract, open-ended, and nontraditional experiment, and so it might not appeal to some people for those reasons. But I loved (and still do) the questions, issues, and hypotheses that motivated the LTEE.

I never thought of the LTEE project as a “lottery ticket.” Maybe I was overly confident, but I was pretty sure the outcomes—whatever they might be—would be cool. I knew enough about what would happen—based on the experiments I had already done—that I was confident the data and analyses would be informative with respect to at least some of my questions. Also, the use of microbes to study evolution in action was still uncommon, so the novelty of the approach would ensure some interest among my colleagues—although let me emphasize that Lin Chao, Dan Dykhuizen, Barry Hall, and Bruce Levin, among others, had already demonstrated the power of using microbes for experimental studies of evolutionary questions.

I should also say, in case it’s not obvious, that I had no idea or intention that the experiment would continue for anywhere near as long as it has lasted. I had previously performed some experiments that lasted several hundred generations, and as I saw the dynamics and thought about the math behind the dynamics, I realized that over those time scales I might be seeing the effects of only one or two beneficial mutations as they swept to fixation. That hardly seemed satisfactory for experiments to explore the structure of the fitness landscape. So I decided the experiment should run for 2,000 generations, over which time I expected there would be at least several fixations of beneficial mutations in each population (and I was right), and that would deserve calling it long-term. That would take a little less than a year, given the 100-fold dilution and 6.6 generations of regrowth each day.

**Fig 1:**
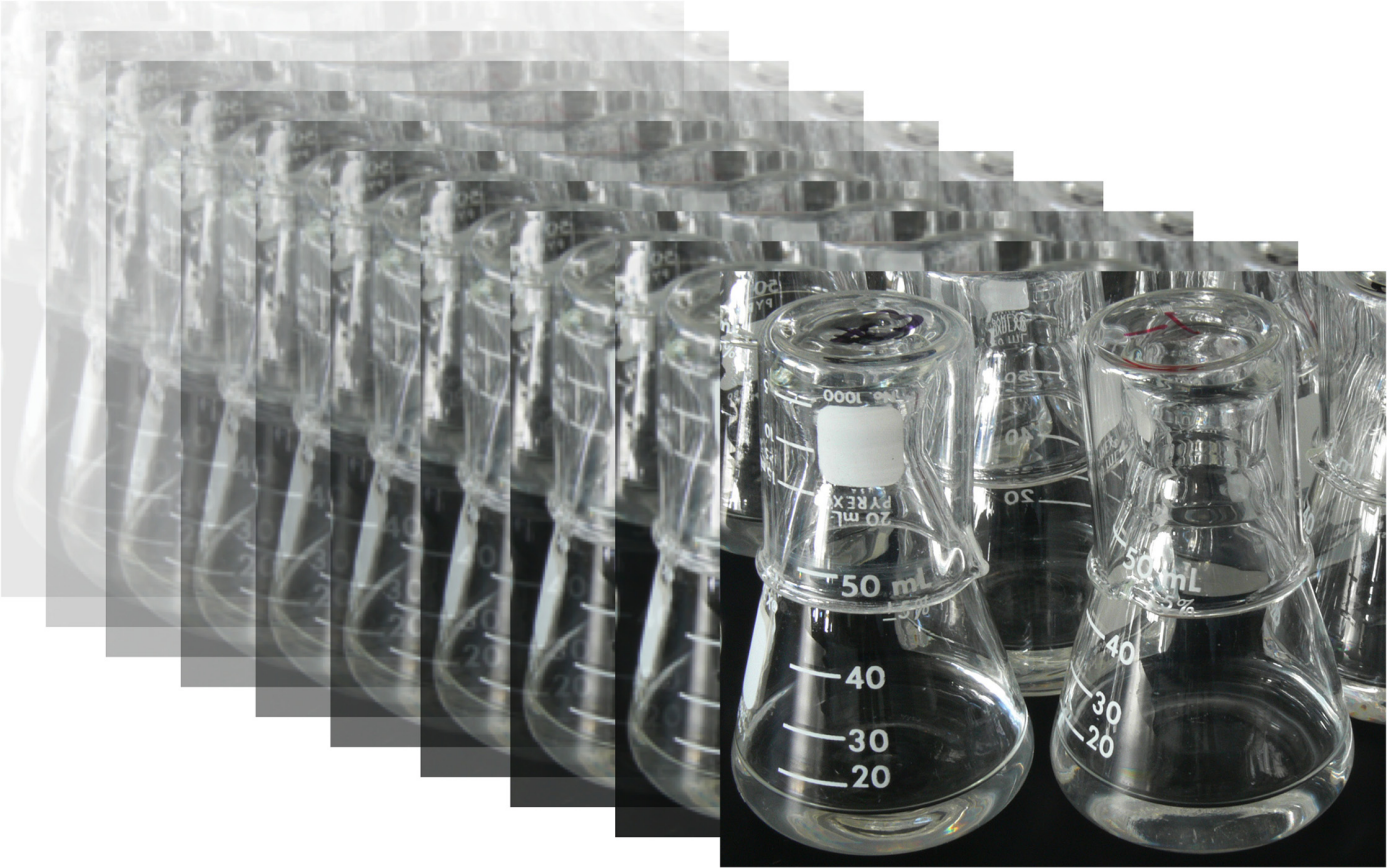
The long-term evolution experiment. The experiment involves daily transfers of 12 *E*. *coli* populations, and it has been running for over a quarter century. *Image credit: Composite image by Richard Lenski and Brian Baer, Michigan State University.*

Of course, propagating the lines for 2,000 generations was one thing—running the competitions to measure fitness, analyzing the data, writing the paper, responding to reviews, all that took longer. So while the experiment began in February 1988, the first paper [1] was published in December 1991. Meanwhile, the generations ticked by (Fig 1). The baseline work of keeping the populations going is not that onerous—yes, somebody has to attend to the transfers every day, but once a lab team reaches a moderate size, it’s not too hard to arrange. And I lived next to the campus in Irvine, so it wasn’t hard for me to come in on the weekends and holidays…my wife still loves me, and my kids recognized my face.

You also wondered whether some of the interesting outcomes had occurred to me when I started. Definitely not! I had made a strategic decision to make the environment of the LTEE very simple to eliminate, or at least reduce, certain complications. And while I think my planning kept these complications from getting out of hand, the tension between the simplicity of the experimental design and all the complications has definitely been part of its interest. I used to complain, mostly in jest, that “Evolutionary biologists say I’m asking the right questions but studying the wrong organism, and microbiologists tell me I’m studying the right organism but asking the wrong questions.” I got that sort of response occasionally, but many people from both fields were very interested and encouraging.

**JF:** I’m interested in your remark about the choice of culture conditions; how they were chosen to maximize the simplicity of the resulting evolution. Microcosm experiments sometimes are criticized as “oversimplified” or “rigged,” the implication being that they’re too simple to yield anything other than the results the experimenter expected. I think your comment is the best refutation of that complaint. You tried to rig it to be simple, but you still got surprised with a much richer and more complicated range of outcomes than you anticipated. As someone who works in microcosms myself, I jokingly tell people, “Believe me, if I could rig microcosms to only give me the answers I anticipated in advance, I totally would!”

**RL:** Thanks, Jeremy. I have seen some experiments proposed that come too close, for my taste, to drawing red and blue marbles from a box to be very interesting. There needs to be some real biology and, as you say, the potential for surprises. And there certainly have been many surprises in the LTEE.

**JF:** Did the LTEE have any hypotheses initially, and if so, how were you going to test them? I ask because, with just one treatment and no a priori model of how the experiment should turn out, it’s not clear to me how it initially could’ve been framed as a hypothesis test. For instance, I don’t see how to frame it as a test of any hypothesis about the interplay of chance and determinism in evolution. It’s hard to imagine getting any result besides some mixture of the two.

**RL:** Yes, the LTEE had many hypotheses, some pretty clear and explicit, some less so. What, did you think I was swimming completely naked?

Before we get to hypotheses, though, I like to begin with general questions about how and why things are the way they are. The LTEE originally set out to answer three sets of questions. First, concerning the dynamics of adaptation by natural selection: is improvement invariably slow and gradual? Or are there periods of rapid change and stasis, even in a constant environment? How long can fitness continue to increase in an unchanging environment? Second, how are phenotypic and genotypic evolution coupled, both dynamically and mechanistically? Third, what about the repeatability of evolution: will replicate populations evolve to the same or different fitness peaks? If phenotypes change in parallel, then does that imply parallel evolution at the level of nucleotides, genes, or pathways?

The first set of questions, about the dynamics of adaptation, had clear expectations that were testable in a fairly standard hypothesis-driven framework. For example, I was pretty sure we would see the rate of fitness improvement decelerate over time, and it has [2]; and I was also pretty sure we’d see a quasi-step-like dynamic to the early fitness increases, and we did [3]. Nonetheless, these analyses have yielded surprises as well, including evidence that fitness can increase indefinitely, and essentially without limit, even in a constant environment [2]. In regard to the second set of questions, about the dynamics of genome evolution and their coupling to phenotypic changes—I’m sure these were part of my original thinking, but I admit that I had almost no idea how I would answer them. Hope sprung eternal, I guess; fortunately, wonderful collaborators, like the molecular microbiologist Dom Schneider, and new technologies—wow, sequencing entire genomes—saved the LTEE.

**JF:** I like your distinction between broad motivating questions and more specific, testable hypotheses. I probably should’ve been clearer in my question that I knew you had the former, but wasn’t sure if you had the latter. So no, I didn’t think you were swimming completely naked! I guess I was wondering more if you were swimming in a Speedo or in one of those Victorian-era full-body swimming costumes.

Your answer here hints at the answer to another of my questions: why not have two or more treatments in the LTEE? Since your hypotheses were about how evolution by natural selection should always work—fitness increases should decelerate, the early phase of all adaptive walks should be step-like—you didn’t need multiple treatments.

Interesting to hear that for some motivating questions you also had specific hypotheses that you knew how to test, while for other motivating questions you didn’t. I wonder, if you hadn’t had specific hypotheses for any of your motivating questions, would the LTEE still have seemed like a good idea? I can imagine that it would have. I think there’s an important role for experiments that are out ahead of theory and hypothesis development, that give theory a “target to shoot at,” something to explain. In my own field of ecology, that’s how I think of the early biodiversity–ecosystem function experiments.

**RL:** We’re thinking along similar lines here. You’ve started to anticipate my answer to your question “Is the LTEE actually an experiment?”

**JF:** Ok, so let me ask you that. Is the LTEE actually an experiment, and wouldn’t it have been even better if it was? It’s just one “treatment”—12 replicates of a single set of conditions. Wouldn’t it have been even more interesting to have, say, two treatments? Two different culture conditions, two founding genotypes, or two founding species?

**RL:** You’re certainly right, Jeremy, that experiments in the fields of ecology and evolutionary biology typically have two or more treatments. But that’s not an essential part of the definition of an experiment. It would have been nice, perhaps, if the LTEE did have two or more environments and/or two or more ancestors, as you suggest—in fact, we’ve run several of those types of experiments over the years, and I’ll mention a few of them below.

The reason I didn’t do that with the LTEE, though, was because one of my core motivating questions concerned the repeatability of evolutionary dynamics across replicate population. That’s a question about the trajectory of variances over time, which is challenging statistically because estimates of variances have large uncertainties. So if the LTEE had two treatments, I might have been able to say something about the differences between them, but I would have had less power to say anything about the among-replicate variances for either treatment. So for that motivating question, going from 12 replicate populations down to 6 replicates would have been risky.

It certainly would be nice to have more total populations, say, 24 or even more; nowadays, many labs use 96-well plates for evolution experiments, with each well a replicate population and robots to automate the transfers. When I started the LTEE, though, we worked with flasks (albeit small ones); 12 may not seem like too many, but when we run the competition assays to measure fitness, we then have replicate assays for each population and we analyze multiple generations simultaneously, so the students and postdocs running these assays are handling many dozens or even hundreds of flasks.

Stepping back a bit, I’d like to suggest that the LTEE is a sort of meta-experiment, to coin a term. By “meta,” I mean the LTEE transcends what one usually considers an experiment because it enables experimentation at several levels.

First, the LTEE is an experiment in the sense that it set out to measure, under defined conditions and with replication, certain quantities, such as fitness trajectories. It may not be typical in having a single treatment, but the temporal dimension coupled with being able to analyze multiple time points simultaneously—that is, the “time travel” enabled by the frozen samples across the generations, including the use of the ancestral strain as an internal control in fitness assays—functions in much the same way from an analysis standpoint.

Second, the LTEE has generated new questions and hypotheses that are amenable to structurally independent follow-on experiments. Let me give two examples. We observed early on that several populations had evolved changes in their DNA metabolism and repair that caused their mutation rates to increase by roughly 100-fold [4]. Such “mutator” mutations can spread by hitchhiking, albeit only occasionally and stochastically, with beneficial mutations that they cause. It wasn’t clear, though, whether they would necessarily increase the rate of fitness improvement, given the large populations and supply of beneficial mutations in the LTEE. So we designed a separate, shorter-duration experiment with 48 populations where we varied the mutation rate, population size, and initial fitness of the founding ancestor, and assessed the fitness gains over 1,000 generations [5].

**Fig 2:**
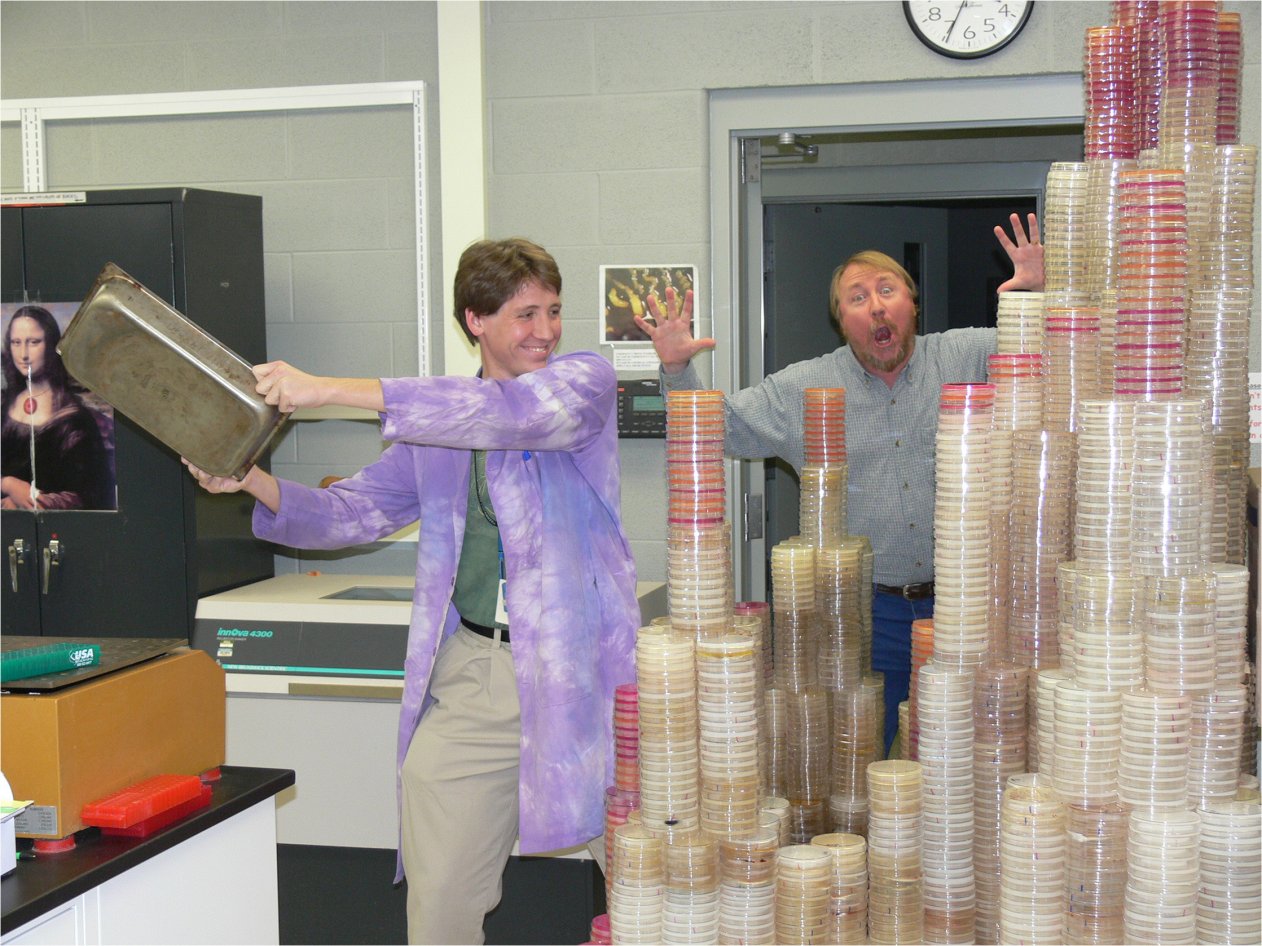
Petri plates aplenty. Former student, now postdoc, Zachary Blount and Richard Lenski horsing around with some of the Petri dishes from Blount's work on the evolution of citrate utilization in one population. *Image credit: Brian Baer, Michigan State University.*

Another case is the “replay” experiments that Zachary Blount ran after one lineage evolved the ability to grow on citrate in the presence of oxygen, which *E*. *coli* generally cannot do [6]. Zack ran thousands of populations (Fig 2) that started from genotypes isolated at different times from the population that eventually evolved this new function, in order to test whether it could have arisen at any time by an appropriate mutation or, alternatively, required first evolving a “potentiated” genetic background, or context, in which the “actualizing” mutation would confer the citrate-using phenotype.

In both of these examples, the subsequent experiments, though separate and distinct from the LTEE, emerged from the LTEE. That is, the questions and hypotheses tested in these experiments were motivated by observations we had made in the LTEE itself.

The third level of the meta-experiment involves questions that arise outside of the LTEE, but for which the LTEE generates materials—specifically, strains—that are useful for experiments to address those questions. Again, I’ll give a couple of examples.

Many ecologists, physiologists, and others are interested in adaptation to specific environmental factors—such as resource availability, temperature, etc.—as well as examining possible tradeoffs associated with adaptation to those factors. One difficulty, though, is that by moving organisms from nature into the lab and allowing them to evolve under, say, different temperature regimes, adaptation to the shared features of the lab environments may outweigh adaptation to the variable of interest. If so, that would interfere with one’s ability to identify the mutations and adaptations most relevant to the factor of interest, and it could obscure tradeoffs that might be important if populations were already adapted to the other aspects of the environment. With these considerations in mind, Albert Bennett and I took a strain from the LTEE that had evolved in and adapted to those conditions, and we used it as the ancestor for a new experiment where six populations evolved under each of four different thermal regimes: 32C, 37C, 42C, and daily alternations between 32C and 42C [7]. In that way, we could focus attention on temperature-specific adaptations, which were Al’s main interest, rather than having such changes overwhelmed by adaptation to the lab environment.

My second example where LTEE-derived strains served as ancestors for an experiment to address an extrinsic question is one of an abstract nature. In this study, we quantitatively partitioned the effects of adaptation, history, and chance on phenotypic evolution by founding 3 populations from 12 different ancestors—each one a genotype sampled from a different one of the LTEE populations—and we then let these 36 populations evolve in a new environment, where we changed the limiting nutrient [8]. By measuring the fitness of the 12 ancestors and 36 derived lines in the new environment, we could disentangle and quantify the relative contributions of adaptation, history, and chance to the observed outcomes. Adaptation measured the tendency for fitness to increase, history reflected the effect of the different starting genotypes on the fitness achieved, and chance the variation in the resulting fitness among the replicates that started from the same ancestor.

**JF:** Thanks for this, Rich! “It’s only one treatment because I needed to estimate variances and so needed lots of replication, and more than 12 replicates wasn’t feasible” is the answer I expected—and it’s an excellent answer. The third level of the meta-experiment is particularly interesting to me. I’d say that is a way of using the LTEE itself as a model system in its own right. *E*. *coli* of course is a model system because we know so much about it and can leverage that knowledge to ask questions that wouldn’t be tractable to ask with other organisms. But the *E*. *coli* in the LTEE are now model organisms for asking questions that arise outside the LTEE, and that wouldn’t be tractable even with “ordinary” *E*. *coli*.

How have you maintained funding for the LTEE over the years, and how hard has it been? The difficulty of sustaining funding for long-term work is a common complaint in ecology, and I’m guessing in evolution as well. And of course, if people think that they won’t be able to sustain funding for a long-term project, they’re less likely to start one in the first place. You’ve already noted that you had some hypotheses initially, and that it wasn’t your initial plan to maintain the LTEE for so long. But when you went back for your first (or second, or third) renewal, presumably you didn’t say, “A bunch of cool stuff has happened already, so please give me more money to keep it going, just to see if anything else cool happens.” So what did you say? Also, has it become easier to get funding to keep it going as you’ve gone along? Has it gotten to the point where the experiment is widely seen as an “institution”? So that people are basically eager to hand you money to keep it going, no questions asked? And looking ahead, how will the LTEE continue after you’re gone?

**RL:** All in all, I’ve been very fortunate with funding for my research. My first attempt to fund the LTEE was rejected, but around that time I received a Presidential Young Investigator Award from the National Science Foundation (NSF) that gave me considerable freedom to pursue the research directions that most interested me. Various grants have supported the LTEE since then including, for the past ten years, an NSF LTREB grant (LTREB stands for long-term research in environmental biology). LTREB grants are very small, but mine provides core support to keep the lines going. Other funds are required to do anything more than some basic quality control and assays. My professorship at MSU provides discretionary funds that allow us to explore new scientific directions and techniques as they become interesting and available, without requiring us to first secure funding. And the graduate students and postdocs in my group have been very talented, and they’ve often been awarded fellowships that fund the brainpower and hard work that has made the LTEE so successful.

Over the years, I’ve taken proposal writing very seriously, emphasizing both the overarching questions that have been with the LTEE since it began and the specific aims that arise from new discoveries and technical advances. One always has to make the case for why a particular project, individual, or team merits support. So I wouldn’t say it has gotten easier to get funding, especially given the decline in funding rates. But I do sense that reviewers have, on balance, become increasingly excited by the LTEE project over the years, as it has borne a lot of fruit. In fact, I’ve just heard that the LTREB grant will be funded again for the next five years. During the pre-proposal phase (yes, a pre-proposal was required for a project that has run for over a quarter century), the panel summary called the LTEE “this community’s Hubble Telescope.” That was certainly gratifying!

The big challenge going forward will be to secure funds that will allow the LTEE to continue after I’m gone. Many colleagues have told me that the LTEE must continue, and I agree. (I’m not planning on retiring anytime soon, but I think it’s wise to hand off a project sooner rather than wait to the last hour.) I like to call the LTEE the experiment that keeps on giving, so the challenge is to find a way to make that happen.

I realize that not every scientist will have the same good fortune that I’ve had. Indeed, by continuing “someone else’s experiment,” a young scientist might even be viewed by some as unoriginal and thus unworthy of the privileges of tenure and funding. To overcome that stigma, I’d like to secure funds to ensure that not only can the LTEE continue but that its continuation is rewarding rather than burdensome to future scientists. After all, it comes with its own inherent challenges—including the fact that the populations are tended every day and management of the ever-growing collection of frozen samples.

My thinking is that each successive scientist responsible for the LTEE would, ideally, be young enough that he or she could direct the project for 25 years or so, but senior enough to have been promoted and tenured based on his or her independent achievements in a relevant field (evolutionary biology, genomics, microbiology, etc.). Thus, the LTEE would continue in parallel with that person’s other research, rather than requiring his or her full effort, just like my team has conducted other research in addition to the LTEE. The goal, then, is to provide future project leaders with the benefits of continuing the LTEE while relieving them of the most onerous burdens.

So as I’ve said before [9], “If you know anyone who would like to endow a million-year experiment, have them get in touch with me.”

## Blog URLs

Dynamic Ecology, http://dynamicecology.wordpress.com

Telliamed Revisited, http://telliamedrevisited.wordpress.com

## Abbreviations:

LTEE: Long-Term Evolution Experiment
LTREB: long-term research in environmental biology
NSF: National Science Foundation
